# Oceanographic Fronts Shape *Phaeocystis* Assemblages: A High-Resolution 18S rRNA Gene Survey From the Ice-Edge to the Equator of the South Pacific

**DOI:** 10.3389/fmicb.2020.01847

**Published:** 2020-08-06

**Authors:** Swan L. S. Sow, Thomas W. Trull, Levente Bodrossy

**Affiliations:** ^1^Institute for Marine and Antarctic Studies, University of Tasmania, Hobart, TAS, Australia; ^2^Oceans and Atmosphere, Commonwealth Scientific and Industrial Research Organisation (CSIRO), Hobart, TAS, Australia

**Keywords:** haptophytes, *Phaeocystis*, Southern Ocean, oceanographic fronts, winter distribution, 18S (SSU) rRNA gene, latitudinal diversity gradient, amplicon sequence variants

## Abstract

The cosmopolitan haptophyte *Phaeocystis* is recognized as a key contributor to marine biogeochemical cycling and important primary producer within polar marine environments. Yet, little is known about its solitary, non-colonial cell stages or its distribution during the colder, low-productivity seasons. We examined the biogeography of *Phaeocystis* along a high-resolution (0.5-degree latitudinal interval) transect from the Antarctic ice-edge to the equator of the South Pacific, in the austral autumn-winter. Using high-throughput 18S rRNA gene sequences with single nucleotide variable (zero-radius) operational taxonomic units (zOTUs) allowed us to explore the possibility of strain-level variation. From water samples within the upper water column, we show the presence of an abundant *Phaeocystis* assemblage that persisted during the colder months, contributing up to 9% of the microbial eukaryote community at high latitudes. The biogeography of *Phaeocystis* was strongly shaped by oceanographic boundaries, most prominently the polar and subantarctic fronts. Marked changes in dominant *Phaeocystis antarctica* zOTUs between different frontal zones support the concept that ecotypes may exist within the *Phaeocystis* assemblage. Our findings also show that the *Phaeocystis* assemblage did not abide by the classical latitudinal diversity gradient of increasing richness from the poles to the tropics; richness peaked at 30°S and declined to a minimum at 5°S. Another surprise was that *P. globosa* and *P. cordata*, previously thought to be restricted to the northern hemisphere, were detected at moderate abundances within the Southern Ocean. Our results emphasize the importance of oceanographic processes in shaping the biogeography of *Phaeocystis* and highlights the importance of genomics-based exploration of *Phaeocystis*, which have found the assemblage to be more complex than previously understood. The high winter relative abundance of the *Phaeocystis* assemblage suggests it could be involved in more complex ecological interactions during the less productive seasons, which should be considered in future studies to better understand the ecological role and strategies of this keystone species.

## Introduction

Climate change is fast altering many aspects of marine ecosystems (Burrows et al., 2011; Achterberg, 2014). Thus, advancing our knowledge on keystone organisms such as phytoplankton, which form the base of marine food webs, is central in forecasting the effects of climate change. The prymnesiophyte phytoplankton of the genus *Phaeocystis* have a cosmopolitan marine distribution and play pivotal roles in the marine carbon and sulfur cycles (DiTullio et al., 2000; Verity et al., 2007). This is particularly true at high latitude marine environments such as the Southern Ocean, where primary productivity has a considerable contribution toward global ocean biogeochemistry and climate (Sarmiento et al., 2004). *Phaeocystis antarctica* is a known species commonly found in the Southern Ocean, where it exhibits extensive blooms (El-Sayed et al., 1983). It has significantly higher rates of carbon dioxide (CO_2_) and nitrate drawdown as well as rapid carbon export potential compared to diatoms (Arrigo et al., 1999; DiTullio et al., 2000). Thus, changes to the *Phaeocystis* community could strongly impact nutrient cycling efficiency and carbon export (Schoemann et al., 2005; Hutchins and Fu, 2017). However, *Phaeocystis* blooms are thought to be prevalent only within high latitude environments during the spring-summer months (Long et al., 2007). Seasonal variation and succession of overall composition of microbial communities is well recognized (Winter et al., 2009; Gilbert et al., 2012; Grzymski et al., 2012; Salter et al., 2015; Parada and Fuhrman, 2017), but little is known about the *Phaeocystis* assemblage outside its blooming season. This is because molecular identification of *Phaeocystis* remains scarce and morphological (microscopy) identification is ambiguous (Baumann et al., 1994). This situation is exacerbated by the complexity of *Phaeocystis* ecology, which includes a polymorphic life cycle with a wide distribution of its solitary free-living form, as well as the occurrence of colonial assemblages in which hundreds of cells are grouped in a polysaccharide matrix (Schoemann et al., 2005). This complexity means that its effective size in the plankton community can vary several orders of magnitude, from a few microns for the solitary forms to a few hundreds of microns for colonies. Current understanding is limited by insufficient temporal and spatial sampling of *Phaeocystis*, along with incomplete understanding of species and strains (Verity et al., 2007).

The full range of controls on the oceanic distribution of *Phaeocystis* species and strains remains to be determined. It is known to be regulated in part by its specific light and nutrient requirements, particularly the concentration of phosphate and nitrate (Verity et al., 2007). Within the Southern Ocean, micronutrients such as iron are limiting and also play a role in controlling *Phaeocystis* distribution (Stefels and van Leeuwe, 1998; van Leeuwe and Stefels, 1998, 2007). Because species and community composition of microbes are known to differ with ocean salinity and temperature (Brown et al., 2012; Raes et al., 2018), it is expected that oceanographic features are likely to be important in the control of *Phaeocystis* distributions, especially fronts which separate the euphotic ocean into zones or regions which have different hydrographical properties such as salinity, temperature, density and nutrients (Sokolov and Rintoul, 2002). For example, the Southern Ocean polar front is known to be a major ecological boundary for bacteria, archaea and zooplankton (Chiba et al., 2001; Hunt et al., 2001; Esper and Zonneveld, 2002; Ward et al., 2003; Wilkins et al., 2013; Raes et al., 2018).

Another expectation is the latitudinal diversity gradient, characterized as an increase in species richness from higher to lower latitudes (Ekman, 1953), as a fundamental biodiversity pattern found across a broad range of terrestrial, aquatic and marine taxa (Pianka, 1966; Gaston, 2000; Hillebrand, 2004). However, knowledge on the applicability of this biodiversity pattern on the highly diverse microbial communities remain uncertain. The gradient effect is expected to decrease with decreasing organism size, as microbes have large population sizes, rapid generation times and are easily dispersed (Finlay, 2002). With the exception of several recent works (Pommier et al., 2007; Fuhrman et al., 2008; Ladau et al., 2013; Endo et al., 2018; Raes et al., 2018; Ibarbalz et al., 2019), the microbial community, particularly microbial eukaryotes such as *Phaeocystis* are still under-represented with regards to research relating to latitudinal diversity patterns. Exceptions to the gradient have also been reported (Brayard et al., 2005; Tittensor et al., 2010; Powell et al., 2012; Woolley et al., 2016).

In the late austral autumn to winter of 2016, we investigated distribution trends of *Phaeocystis* from high to low latitude (−66 to 0°) epipelagic waters using high-throughput sequencing of the 18S ribosomal RNA marker gene. Our research encompassed both colonial and non-colonial *Phaeocystis* assemblages sampled on a latitudinal transect spanning ∼ 7000 km within the South Pacific Ocean, using the first high-resolution (∼0.5° sampling interval) study. Our goal was to refine our understanding of *Phaeocystis* distributions outside the period of its summer blooms. We hypothesized that stable fronts and oceanographic features can act as ecological boundaries that delineate the beta-diversity patterns of the *Phaeocystis* assemblage. From this hypothesis, we also set out to examine the species composition within each zone during the austral autumn-winter. This included determining whether the *Phaeocystis* assemblage followed the latitudinal diversity gradient during the less-productive seasons.

## Materials and Methods

### Study Region and Sampling

Seawater samples were collected on the RV *Investigator* during the GO-SHIP P15S repeat hydrographic transect^1^ (26th April to 29th June, 2016). Seawater was collected on a 36-bottle rosette water sampler mounted with the SBE911 conductivity, temperature and depth (CTD) sensors (Seabird Scientific, United States), SBE43 dissolved oxygen sensor (Seabird Scientific, United States), Aquatracka fluorometer (Chelsea Technologies, United Kingdom) and Wet Labs C-Star^TM^ transmissometer (Seabird Scientific, United States).

**FIGURE 1:**
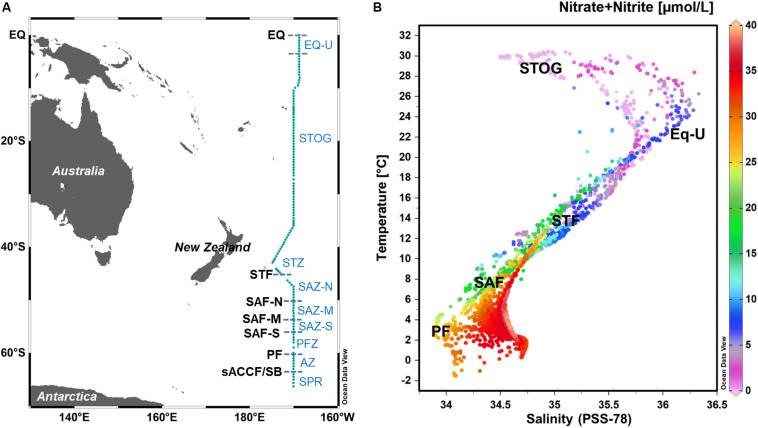
**(A)** Map depicting microbial sampling stations within the GO-SHIP P15S transect (teal line) with approximate locations (latitudes) of the major oceanic fronts. **(B)** Plot of temperature against salinity (T-S) overlaid with nitrate + nitrite (NOx), with indicative temperature-salinity-NOx ranges of the different oceanic zones. T-S plots were generated using data recorded from all depths measured within the full water column. EQ, equator; EQ-U, equatorial upwelling zone; STOG, subtropical oligotrophic gyre zone; STZ, subtropical zone; SAZ-N, subantarctic zone-north; SAZ-M, SAZ-mid; SAZ-S, SAZ-south; PFZ, polar frontal zone; AZ, Antarctic zone; sACCF, southern Antarctic circumpolar current front; SPR, subpolar region.

Samples for DNA analysis of the *Phaeocystis* assemblage were collected from 10-litre Niskin bottles deployed at four different depths within the upper water column (depth ranges 1–286 m; see Supplementary Table S1). The four sampling depths within the upper water column consisted of one sample each from the surface, deep chlorophyll maximum, mixed layer depth (MLD) and just below the mixed layer, and covered a latitudinal range of 0–66°S at longitude ranges of 169–174°W (Figure 1). Water column mixed layer depths were calculated according to Talley et al. (2011). For MLD values along the P15S transect referenced in this study, see Supplementary Figure S5 in Raes et al. (2018).

### Physico-Chemical Variable Measurements and Nutrient Analyses

Hydrochemistry and nutrients analyses were assayed by the Commonwealth Scientific and Industrial Research Organisation (CSIRO) Hydrochemistry team as described in Raes et al. (2018). In this paper, all presented salinities are based on the PSS-78 reference (S_P_) and are therefore unitless. The latitude, longitude, and absolute pressure values at the depths of sample collection (i.e., the sample “depths” in pressure units of dbar) provided with the data enable Absolute Salinity (S_A_) to be calculated via the TEOS-10 equation^2^ of state for seawater. Chlorophyll *a* profiles were generated using extracts from 0.525 liters of sample water from five sampling depths within the upper water column. Extraction was done using gentle vacuum filtration (pressure drop < 10 kPa) using 25-mm GF/F grade Whatman^®^ glass microfiber filters (Merck, Germany) and samples were measured on a Trilogy laboratory fluorometer (Turner Designs, United States). Chlorophyll *a* data is as presented in Raes et al. (2017). Physical, biogeochemical, nutrient and metadata reported or discussed here can be accessed through the CLIVAR and Carbon Hydrographic Data Office (CCHDO) webpage^3^.

### DNA Isolation, Amplification and High-Throughput Sequencing

Two liters of seawater were collected for each sample, filtered through 0.22 μm pore size polyethersulfone Sterivex^TM^ filter cartridges (Millipore, Germany) and stored immediately at −80°C until DNA extraction. DNA isolation was performed using a modified organic (phenol:chloroform:isoamyl alcohol-based) DNA isolation protocol of the DNeasy^®^ PowerWater^®^ Sterivex^TM^ Kit (Qiagen, Germany) (Appleyard et al., 2013; Supplementary Methods). Individual microbial samples were assigned unique Bioplatforms Australia (BPA) IDs from the Australian National Data Service (Supplementary Table S1). DNA was sequenced at the Ramaciotti Centre for Genomics^4^ (University of New South Wales, Sydney), where amplicons targeting the V4 region of eukaryotic 18S rRNA gene [18SV4F and 18SV4R primer pair modified from Stoeck et al. (2010), see Supplementary Table S7] were generated and sequenced using the MiSeq^TM^ (Illumina, United States) dual-indexed 250 bp paired-end approach following protocols established by the Australian Microbiome (Marine Microbes) Initiative^5^. Full PCR amplification conditions, primer sequences and sequencing protocols used can be found in Supplementary Methods or downloaded from the methods page of the Bioplatforms Australia data portal^6^.

### Bioinformatics, Statistical and General Analyses

All sequences were analyzed as part of the Australian Microbiome Initiative as previously described by Brown et al. (2018) (Supplementary Methods). Zero-radius operational taxonomic unit (zOTU) data with single nucleotide variation between zOTUs were used to enable data analysis at the highest possible phylogenetic variation (Edgar, 2016, 2018). zOTUs were taxonomically classified using the Protist Ribosomal Reference Database (PR^2^, v4.11.1) (Guillou et al., 2013), and zOTU tables were subsampled to a constant sampling depth of 25,000 sequences per sample for subsequent statistical analyses using the “sub.sample” command in MOTHUR v.1.36.1 (Schloss et al., 2009). zOTUs from all samples that were assigned to the family Phaeocystaceae (and genus *Phaeocystis*) were considered within this study.

Paired end reads (R1, R2), indexed reads (I1, I2) in.fastq format, and latest sequence read abundance tables are available from the Australian Microbiome page of the Bioplatforms Australia data portal^7^. Genomic datasets associated with this study are accessible from the NCBI BioProject PRJNA385736 webpage^8^. Sequence accession numbers for each sample are listed in Supplementary Table S1.

Biogeographic distribution patterns are known to be influenced by sampling effort (number of sequences) (Chaudhary et al., 2017; Meyer et al., 2018; Hermans et al., 2019). Lower abundance or sporadically present zOTUs may be present as a result of sequencing artifacts or noise, affecting the credibility of inferred biogeographic information. To account for this, we considered the abundance-ubiquity (Ab-Ub) relationship when analyzing relative abundances of the *Phaeocystis* assemblage. The Ab-Ub relationship of all *Phaeocystis* zOTUs were computed using Perl scripts from the CORe microBiome Analysis Tools (Corbata) project (Li et al., 2013). We considered 0.01% of the cumulative relative abundance (relative abundance averaged across all the samples analyzed) and 20% ubiquity as the minimum Ab-Ub threshold (which would translate to 2.5 sequencing reads and 25 sampling sites, respectively).

Richness and Good’s coverage for the *Phaeocystis* assemblage was calculated with 100 permutations using the “ecopy” (Lemoine, 2015) and “scikit-bio” packages on Python 3.6.1. Rarefaction curves were generated using the “rarecurve” function in the R package “vegan” (Oksanen et al., 2018). Richness, Good’s coverage and rarefaction curves were calculated based on average zOTU relative abundance values of the four sampling depths within the upper water column per station (latitude), to obtain single values reflecting the overall *Phaeocystis* assemblage within the upper water column. Richness was defined as the number of zOTUs (non-zero columns) observed per station (“spRich” function from “ecopy”). Good’s coverage was defined as C = 1−(n^1^/N), where C is the Good’s coverage estimator, n^1^ is the number of zOTUs that have been sampled once and N is the total number of individuals in the sample (“skbio.diversity.alpha.goods_coverage” function from “scikit-bio”). Analysis of similarities (ANOSIM) was conducted in PRIMER (v7.0.13, PRIMER-E, United Kingdom) (Clarke and Gorley, 2015) following square-root transformation of zOTU abundances, to test for the strength of assemblage clustering according to oceanic zones.

The relative abundance and alpha-diversity distribution of the *Phaeocystis* population (after accounting for low Ab-Ub zOTUs) were analyzed by considering (in separate plots) the percentage of *Phaeocystis* sequences and richness across the range of latitudes sampled. Percentage of *Phaeocystis* sequences were calculated as average *Phaeocystis* relative abundance value per station (of the four sampled depths within the upper water column) against all microbial eukaryote 18S rRNA gene sequences per station (i.e., 25,000 sequences).

Each individual *Phaeocystis* zOTU was categorized as either “high,” “mid” or “low” abundance zOTUs based on the total cumulative percentages for each zOTU of the total *Phaeocystis* sequences. The latitudinal distribution of each *Phaeocystis* zOTU from the “high” and “mid” categories were visualized on a plot of percent relative abundance of each zOTU against latitude. Percent relative abundance of each zOTU was defined by considering its relative abundance (total reads) at each latitude out of total 18S rRNA gene sequence reads per latitude (i.e., 25,000).

A phylogenetic tree was inferred using the neighbor-joining method with 1000 bootstraps using ARB (v6.06) (Ludwig et al., 2004) based on 382 bp aligned regions of the 18S rRNA gene sequences. Sequences were aligned using MUSCLE v3.8.31 (Edgar, 2004) based on the default alignment mode and was rooted using outgroup sequences *Prymnesium parvum* (NCBI accession no.: AJ246269), *Imantonia rotunda* (AJ246267), *Pleurochrysis carterae* (AJ246263), *Gephyrocapsa oceanica* (AB058360), and *Isochrysis galbana* (AJ246266).

Most analyses and graphical plots were generated in the R statistical computing platform (v3.4.4) (R Core Team, 2018) using ggplot2 (Wickham, 2016), Microsoft Excel and Ocean Data View (v5.1.7) (Schlitzer, 2019).

## Results

### Physico-Chemical Profile and Major Epipelagic Oceanic Zones

**FIGURE 2:**
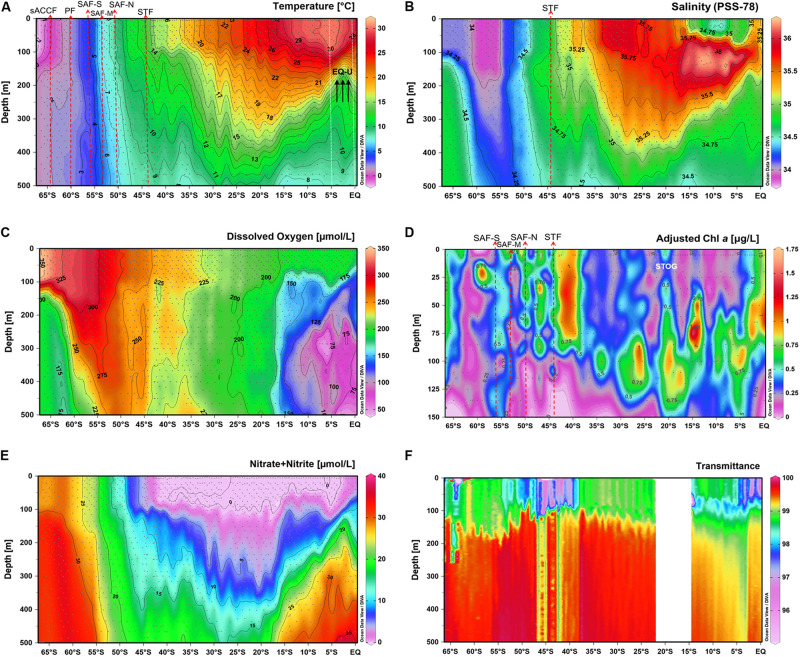
Vertical distributions of **(A)** temperature, **(B)** salinity, **(C)** dissolved oxygen, **(D)** chlorophyll *a*, **(E)** nitrate + nitrite and **(F)** transmittance (%) within the top 500 m of the water column along the P15S sampling section (top 150 m for chlorophyll *a*). Major oceanographic fronts are indicated by red dashed arrows, white dotted lines indicate approximate boundaries for subtropical gyre and equatorial upwelling zones. Front acronyms are as detailed in Figure 1.

From the 132 sampling stations along the GO-SHIP P15S transect, we identified six different ocean fronts and nine different oceanic zones (regions) according to temperature, salinity and nutrient values (Figures 1, 2 and Supplementary Table S1). Fronts and oceanic regions were determined according to temperature and salinity range indicators suggested by Orsi et al. (1995), Sokolov and Rintoul (2002), and Longhurst (2007). Clear temperature (polar front – PF, ∼60°S; subantarctic front – SAF) and salinity (subtropical front – STF, ∼44°S) changes were observed when passing through the fronts (Figure 2). Three branches of the subantarctic front were also observed: SAF-south (SAF-S, ∼56°S), SAF-mid (SAF-M, ∼54°S) and SAF-north (SAF-N, ∼50°S) (Figure 2A). Colder waters at depths of ∼150 m approximately 5°S from the equator were indicative of an equatorial upwelling (Figures 2A,E).

Temperature of the surface waters ranged from −2°C to 30°C and increased gradually from 66°S to the equator (Figure 2A). Salinity values (PSS-78) ranged from 34 to 36.5, were lowest at higher latitudes and increased toward the equator, peaking in subtropical waters at ∼30°S (Figure 2B). Oxygen values of the surface waters peaked at ∼350 μmol L^–1^ at higher latitudes and decreased toward warmer waters at the equator to ∼200 μmol L^–1^ (Figure 2C), consistent with temperature control of oxygen solubility and near atmospheric equilibrium in surface waters. Within the top 50 m of the water column, chlorophyll *a* values were highest (0.75–1.25 μg L^–1^) between 40 and 55°S within the STZ and SAZ, and lowest (0–0.35 μg L^–1^) within the subtropical oligotrophic gyre (STOG) (Figure 2D).

### Richness Profile of the *Phaeocystis* Assemblage Within the South Pacific

The subsampled 18S rRNA zOTU table with samples from the four different sampling depths within the upper water column yielded 237,016 sequences that classified under the *Phaeocystis* genus, which translated to ∼2% of all 18S rRNA gene sequences considered. When considered based on average relative abundance values of the four depths per station (latitude) basis, there was a total of 59,736 sequences from the 132 sampling stations. Rarefactions plots (Supplementary Figure S1) and Good’s coverage of >0.9 for all stations (Supplementary Table S2) indicate adequate sampling to capture the *Phaeocystis* diversity that was present.

A total of 92 *Phaeocystis* zOTUs were observed. We considered the Ab-Ub relationship of all 92 *Phaeocystis* zOTUs found within this study (Supplementary Figure S2), where 29 *Phaeocystis* zOTUs had abundances and ubiquity above the 0.01–20% Ab-Ub threshold (Supplementary Figure S2 and Supplementary Table S3). *Phaeocystis* zOTUs below the Ab-Ub threshold are listed in Supplementary Table S6 with their respective relative abundances.

**FIGURE 3:**
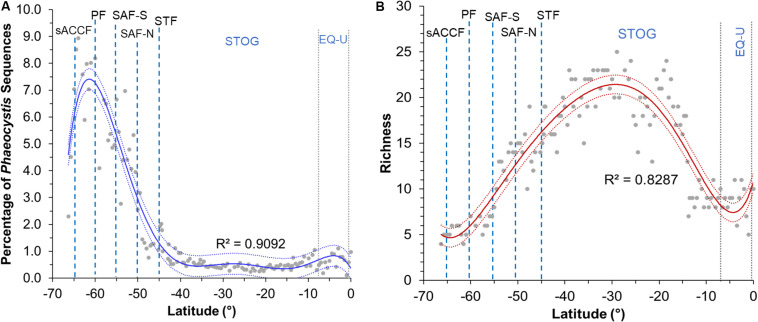
Plot of **(A)** percentage of *Phaeocystis* sequences against latitude and **(B)** richness against latitude for sampling stations across all latitudes. Percentage values presented in plot A are calculated as average *Phaeocystis* relative abundance value per station (of the four sampled depths within the upper water column) against all microbial eukaryote 18S rRNA gene sequences per station (i.e., 25,000 sequences). Sixth-order polynomial fit is indicated by the blue line for the sequence percentage plot and by the red line for the richness plot. 95% confidence intervals are indicated by blue (*Phaeocystis* sequence percentage plot) and red dotted lines (richness plot). Blue dashed lines indicate approximate location of fronts, gray dotted lines indicate approximate boundaries for subtropical gyre and equatorial upwelling zones. Acronyms for fronts and zones are as listed in Figure 1.

**FIGURE 4:**
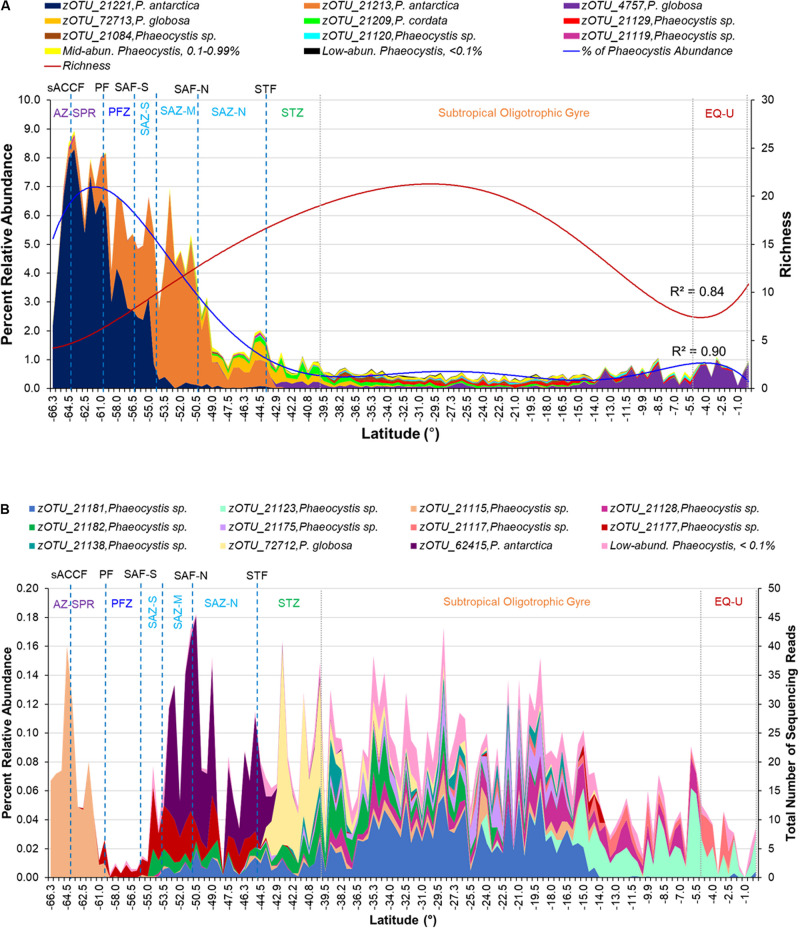
**(A)** Percent relative abundance of high-abundance *Phaeocystis* zOTUs (relative abundance >1% of all *Phaeocystis* 18S rRNA gene sequences) above the Ab-Ub threshold within the P15S transect. Mid- (0.1–0.99% of all *Phaeocystis* 18S rRNA gene sequences) and low- (< 0.1% of all *Phaeocystis* 18S rRNA gene sequences) abundance zOTUs are indicated as separate, pooled categories. Richness (red line) and percent relative abundance (blue line) profiles of the *Phaeocystis* assemblage fitted with a sixth-order polynomial regression curve are overlaid. **(B)** Average relative abundance of mid-abundance *Phaeocystis* zOTUs above the Ab-Ub threshold. Low-abundance zOTUs are indicated as a pooled category. Approximate locations of fronts are indicated by blue dotted lines, while approximate boundaries for subtropical gyre and equatorial upwelling zones are indicated by gray dotted lines. Acronyms for fronts and zones are as listed in Figure 1.

The average percentage of *Phaeocystis* sequences per station within the microbial eukaryote community was greater at the higher latitudes, peaking at ∼9% at 64°S (Figure 3A). Average percentage of *Phaeocystis* sequences (aggregated value for four samples in the water column) decreased toward the STF and was lowest within the subtropical oligotrophic gyre (10–40°S; 0.3–0.9%), increasing slightly again after 5°S within the equatorial upwelling zone (Figure 3A). *Phaeocystis* richness peaked at 25 zOTUs at ∼30°S (*r*^2^ = 0.83) (Figure 3B). Richness increased after crossing the PF (northward) toward the SAF. Richness was lowest in the region right before the PF (∼61°S, four zOTUs) and the equatorial upwelling (∼5°S, six zOTUs) (Figure 3B). Overall, the region with the highest richness corresponded with the region with the lowest percentage of *Phaeocystis* in the subtropical oligotrophic gyre (∼20–40°S) (Figures 3, 4A).

### *Phaeocystis* Assemblage Composition From the Subpolar Region to the Equator

Of the 29 *Phaeocystis* zOTUs above the Ab-Ub threshold, nine (31%) were high-abundance (making up more than 1% of the *Phaeocystis* 18S rRNA gene sequences across all the samples analyzed), and were classified as *P. antarctica*, *P. globosa, P. cordata*, and unclassified *Phaeocystis* (Figure 4A and Supplementary Table S3). Eleven (38%) zOTUs were classified as mid-abundance (0.1–0.99% of *Phaeocystis* 18S rRNA gene sequences) and comprised of *P. antarctica*, *P. globosa* and unclassified *Phaeocystis* species (Figure 4B and Supplementary Table S3). The remaining nine (31%) were low-abundance (< 0.1% of *Phaeocystis* 18S rRNA gene sequences) and were all unclassified *Phaeocystis* (Supplementary Table S3). The 63 *Phaeocystis* zOTUs below the Ab-Ub threshold identified as *P. pouchetti, P. jahnii, P. globosa*, and *P. antarctica* and unclassified *Phaeocystis* (Supplementary Table S6).

Overall, the *Phaeocystis* assemblage was significantly different between most of the oceanographic zones as demonstrated by the ANOSIM test (Supplementary Table S4). Pairwise ANOSIM between STZ and STOG *Phaeocystis* assemblages as well as between STOG and EQ-U assemblages found that *Phaeocystis* in these oceanic zone pairs were moderately but significantly different (*R* = 0.505–0.514, *p* = 0.01; Supplementary Table S4). Assemblages in the PFZ and SAZ-S pair as well as SAZ-S and SAZ-M pair were different (*R* = 0.6), but without statistical significance (*p* = 0.8–2.4; Supplementary Table S4).

The two major *P. antarctica* zOTUs (zOTU_21221, zOTU_21213) were abundant mainly at the higher latitudes within the Southern Ocean sector of the transect (Figure 4A). From the ice edge, *P. antarctica* was abundant beyond the PF up till the SAF, with distinct assemblage composition (zOTU) changes observed after crossing the PF and SAF-M (Figure 4A). At ∼49°S within the SAZ-N, *P. antarctica* declined sharply in relative abundance with a corresponding moderate increase in the relative abundance of *P. globosa* (Figure 4A). The EQ-U zone comprised mainly of *P. globosa*, though the assemblage (zOTUs) were distinct from those found within the SAZ-N and STZ (Figure 4A).

The mid- to low-abundance *Phaeocystis* assemblage also exhibited distinct assemblage compositions between the different oceanographic zones. Notably, the AZ-SPR consisted mainly of an unclassified *Phaeocystis* zOTU (zOTU_21115) not prevalent in other zones, as did the SAZ (Figure 4B). A highly diverse *Phaeocystis* assemblage with higher proportions of low-abundance *Phaeocystis* was observed within the subtropical oligotrophic gyre which had the lowest percentages of *Phaeocystis* sequences (Figures 3, 4).

Latitudinal distribution patterns of *Phaeocystis* relative abundance and richness were consistent whether considering only those above the Ab-Ub threshold or all *Phaeocystis* zOTUs. Latitudinal distribution patterns and ANOSIM incorporating all *Phaeocystis* zOTUs, including those below the Ab-Ub threshold are provided in Supplementary Figure S3 and Supplementary Table S5.

**FIGURE 5:**
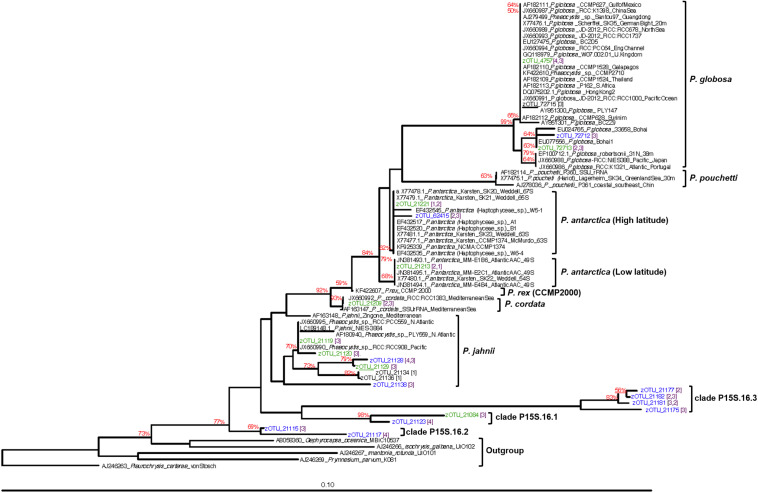
Neighbor-joining tree of abundant *Phaeocystis* zOTUs clustered against other known *Phaeocystis* 18S rRNA gene sequences. Sequences in green are high-abundance (>1% relative abundance) zOTUs, while sequences in blue are mid-abundance (0.1–0.99% relative abundance) zOTUs from this study. Bootstrap values above 50% are indicated at the branches in red. To more easily identify the regions where our *Phaeocystis* zOTUs originated from, we established the following four categories of regions – (1) Antarctic (65–55°S); (2) subantarctic (55–45°S); (3) mid-latitudes (45–15°S); (4) low-latitudes (15°S–0°). Numbers in purple and square parenthesis indicate the region the zOTU was detected. Where more than one number is indicated, the first number indicates the region where higher abundances of that zOTU was detected.

The phylogenetic relationship amongst all high- and mid-abundance *Phaeocystis* sequences from this study with reference to sequences from previous studies are depicted in Figure 5. Known colony-forming species, *P. antarctica*, *P. globosa*, *P. pouchetti*, and *P. cordata* clustered together within a common clade. *P. antarctica* sequences branched out further forming separate high-latitude and low-latitude clusters (Figure 5). *P. antarctica* zOTU_21221 was abundant within the AZ-SPR and PFZ. zOTU_21221 sequences clustered together with *P. antarctica* sequences previously isolated from high latitude (63–65°S) locations at the Weddell Sea and McMurdo Sound (Medlin et al., 1994) which we term the high-latitude *P. antarctica* cluster within this study (Figure 5). *P. antarctica* zOTU_21213 was abundant within the SAZ. zOTU_21213 sequences clustered with sequences previously isolated from a lower latitude (49°S) location at the Atlantic sector of the Antarctic Circumpolar Convergence (ACC) (Zingone et al., 2011) and we term this as the low-latitude *P. antarctica* cluster (Figure 5). zOTU_21120 and zOTU_21119, detected within the subtropical oligotrophic gyre, clustered together with *P. jahnii*. The two *P. globosa* zOTUs (zOTU_72713 and zOTU_4745) that were detected also seemed to group with two distinct subclades on the phylogenetic tree and were each detected in higher abundances in two different regions (subantarctic region for zOTU_72713, low latitude region for zOTU_4745). The remaining unclassified *Phaeocystis* zOTU sequences clustered together to form three separate clades distinct from other reference *Phaeocystis* sequences and clades (clade P15S.16.1, P15S.16.2, P15S.16.3; Figure 5).

## Discussion

While numerous reports have documented *Phaeocystis* as a ubiquitous phytoplankton within the Southern Ocean and southern hemisphere during its peak blooming seasons (Arrigo et al., 1999; DiTullio et al., 2000, 2003; Smith et al., 2000; Vogt et al., 2012; Eriksen et al., 2018), there is an evident lack of studies dedicated to understanding the ecology and distribution of this prevalent phytoplankton throughout the rest of the year (i.e., austral autumn-winter). Most studies are also localized around small latitudinal ranges and centered around the colonial stages of *Phaeocystis*, e.g., Arrigo et al. (1999), Alderkamp et al. (2012), Mills et al. (2012), Delmont et al. (2014). There has been an increasing number of studies utilizing various techniques (microscopy, algal pigments, marker genes) to investigate the global marine plankton [e.g., de Vargas et al. (2015), Swan et al. (2016), Carradec et al. (2018), Righetti et al. (2020)] and *Phaeocystis* (Vogt et al., 2012) distributions. de Vargas et al. (2015) and Carradec et al. (2018) found significant numbers of *Phaeocystis* through gene analysis in the TARA Oceans Expedition, while Estrada et al. (2016) in the Malaspina Expedition did not identify sufficient numbers of *Phaeocystis* to be identified by microscopy. While the global CHEMTAX (Mackey et al., 1996) database established by Swan et al. (2016) analyzed phytoplankton seasonal climatologies, the method fell short due to the variable pigment composition of *Phaeocystis* species (van Leeuwe and Stefels, 1998; van Leeuwe et al., 2014). Despite the advent of global *Phaeocystis* and phytoplankton distribution reports and databases, *Phaeocystis* investigations remain under-represented especially in higher latitudes of the Southern Hemisphere [e.g., Vogt et al. (2012), Endo et al. (2018), Righetti et al. (2020)] and were also biased toward the summer in many cases.

*Phaeocystis antarctica* has previously been shown to harbor physiological adaptations to successfully survive the cold, extreme conditions of austral winters (Tang et al., 2009). Our study corroborates this, as demonstrated by a high proportion of *P. antarctica* relative to the rest of the eukaryote community (up to ∼ 8.6%) across multiple latitudes, showing *Phaeocystis* to be a major phytoplankton in high latitudes during the autumn-winter months as well. During the voyage, a *Phaeocystis* bloom was also opportunistically sighted at approximately 56°S and visually confirmed under the microscope from ship underway samples, suggestive of an active *Phaeocystis* assemblage (Raes et al., 2018). Blooms were not consistently sighted throughout and south of the subantarctic zone of this transect, but high relative abundance of *Phaeocystis* 18S rRNA gene sequences (up to 9% of the microbial eukaryotes per sample) within this region suggests dominance in either colonial and/or solitary cell forms that persist during the colder austral months. Furthermore, 18S rRNA gene sequence of the most abundant *P. antarctica* zOTU (zOTU_21221) showed a 100% sequence identity to an abundant, high-latitude *P. antarctica* 18S rRNA gene sequence detected during a summer Southern Ocean transect close to the Totten Glacier (Sow et al., unpublished). This suggests that what we are observing is the same summer *P. antarctica* assemblage harboring cold-adaptation strategies to maintain abundance over the colder months as opposed to succession by a different autumn-winter *P. antarctica* clade.

Our dataset also clearly demonstrates that oceanographic fronts, particularly the polar front and subantarctic front were strong ecological boundaries that structured the *Phaeocystis* assemblage composition. Previous studies have reported variable responses amongst *P. antarctic*a clones to iron and light stress (Luxem et al., 2017). As environmental conditions vary substantially between frontal zones (Figure 2), the marked change in the dominant *P. antarctica* zOTU north of the SAF-M suggests that the concept of varying ecotypes of *P. antarctica* may exist as previously suggested (Lange et al., 2002; Medlin and Zingone, 2007), with differing nutrient, micronutrient or physicochemical requirements amongst ecotypes. This is supported by the clustering of zOTU_21221 with *P. antarctica* reference sequences previously isolated from high-latitude environments, while zOTU_21213 clustered with *P. antarctica* isolated from a lower latitude, subantarctic region (Figure 5). The distinct increase in abundance of *P. antarctica* zOTU_21213 over *P. antarctica* zOTU_21221 north of the PF followed by the dominance of *P. antarctica* zOTU_21213 past SAZ-M (Figure 4A) suggests that the PFZ may be a transition zone with environmental conditions that are in between optimal conditions for the co-existence of the two different *P. antarctica* zOTUs. This delineation of *Phaeocystis* by oceanographic features was not limited to its most dominant species, since the mid- to low-abundance assemblage also displayed distinct patterns of frontal delineation (Figure 4B).

Our previous work showed that microbial eukaryote richness peaked at the least productive tropical oligotrophic region and that there was a high presence of *Phaeocystis* in the Southern Ocean and STF region (Raes et al., 2018). This study showed that *Phaeocystis* diversity and distribution was in-line with those observed of the overall epipelagic microbial eukaryotes in the P15S line. With several exceptions, the latitudinal diversity gradient (i.e., increased species richness from higher to lower latitude environments) have been thought to apply to most major groups of terrestrial and surface marine taxa (Pianka, 1966; Gaston, 2000; Hillebrand, 2004; Ibarbalz et al., 2019). However, a recent re-evaluation has challenged this (Chaudhary et al., 2016), and there is still debate on whether the theory applies to smaller eukaryotes and prokaryotes (Tittensor et al., 2010). Our findings showed that *Phaeocystis* species richness did not follow the typical latitudinal diversity gradient – richness increased northwards from the pole up to the STOG zone, but peaked at ∼30°S and decreased after that to its lowest point at ∼5°S within the EQ-U zone. This was similar to the trend reported by Chaudhary et al. (2016), who found a dip in species richness close to the equator for a large majority of the 65,000 marine species they re-examined. On the contrary, Ibarbalz et al. (2019) in their recent study of global marine plankton diversity trends based on the Tara Oceans dataset observed the more classical latitudinal diversity gradient of increased species richness toward the equator (mainly attributed to increasing ocean temperatures). They did not observe the dip in species richness close to the equator, though we note that their study examined haptophytes in general and that the latitudinal diversity gradient trend might have been influenced by significant under-sampling of the mid to high latitudes in the southern hemisphere part of their dataset (Ibarbalz et al., 2019). Endo et al. (2018) reported a relatively constant diversity of haptophytes (with large proportions of *Phaeocystis*) across high to low latitudes in the Pacific (i.e., a gradual diversity gradient). However, their study examined diversity gradients of haptophytes as a whole (which may not reflect the exact trend of *Phaeocystis*), and the diversity gradient trends were observed mostly in the northern hemisphere on summer transects.

The increase in richness from polar waters northward to 30°S was completely reversed between 30°S and the equator, but the reversed (decrease) in richness occurred without any change in *Phaeocystis* proportion within the eukaryote population (Figures 3, 4). This suggests that the latitudinal diversity gradient is not monotonic, in contrast to surface temperature (Figure 2A) and thus other factors such as upwelling of nutrients (e.g., NOx; Figure 2E) are likely to be influential.

We also observed a distinct pattern of few winners (low richness) in regions with a thriving assemblage (i.e., high relative abundance at the higher latitudes) and high diversities (high richness) in regions with a struggling assemblage (low abundance) (Figure 4). This could be explained by several theories. *Phaeocystis* blooms require the support of nutrients (van Boekel et al., 1992). Nitrate and phosphate are enriched at higher latitudes (Levitus et al., 1993), which could explain the general prevalence of blooms at high latitudes (Long et al., 2007). In contrast, low nitrate levels within mid-low latitude environments do not encourage bloom formation, and it is likely that most *Phaeocystis* within this region would be free-living. Blooms (colonial forms) of *Phaeocystis* are less vulnerable to predation and viral lysis (Hamm et al., 1999; Hamm, 2000). This suggests it would be more likely for high-latitude *Phaeocystis* (e.g., *P. antarctica*) with adequate nutrient support for bloom formation to be less liable to predation, hence able to proliferate to high relative abundances (note that this interpretation assumes the iron availability is sufficient to allow *Phaeocystis* bloom formation, even though it is considered to be the limiting nutrient for overall phytoplankton productivity in the Southern Ocean). In contrast, mid to low latitude *Phaeocystis*, likely in free-living form, are more liable to viral predation. Hence, it is likely that *Phaeocystis* inhabitants here are prevented from increasing to high abundances even if higher temperatures within the lower latitude regions may suit a wider range of *Phaeocystis* species (i.e., increased richness). Viral predation also appears to be strain specific, with previously isolated strains found to infect only *P. globosa* (Schoemann et al., 2005) and *P. pouchetti* (Bratbak et al., 1998). Interestingly, besides recent reports of a virus predicted to be capable of infecting *P. antarctica* (Alarcon-Schumacher et al., 2019), no virus confirmed to infect *P. antarctica* has been isolated thus far. Finally, less species can tolerate the extreme environmental conditions (e.g., low temperatures) or increased variation of climatic conditions at higher latitudes (i.e., climate harshness/stability hypothesis) (Currie et al., 2004). This may translate to reduced competition at higher latitudes (Amend et al., 2013), allowing surviving species (e.g., *P. antarctica*) to flourish/dominate. The *Phaeocystis* genus, particularly in its free-living form, is known to have a cosmopolitan distribution but exists as distinct species across diverse marine environments (Baumann et al., 1994; Schoemann et al., 2005). As observed within our study, *P. antarctica* is the prevailing species within high latitude southern hemisphere waters (Figure 4 and Supplementary Table S3) given it has available adaptation strategies to lower temperatures (Medlin et al., 1994). However, it is interesting that *P. globosa*, while not dominant, was detected at moderate abundances within the southern SAZ and STF (Figure 4). While *P. globosa* has been observed in temperate and tropical waters (Chen et al., 2003; Schoemann et al., 2005; Rousseau et al., 2013), its occurrence has, to the best of our knowledge, never been reported within temperate waters of the southern hemisphere. The detection of *P. cordata* within southern SAZ and STF (Figure 4) is also surprising, as this is a species that has also thus far only been detected within northern hemisphere waters, first in the Mediterranean Sea (Zingone et al., 1999) and later also in parts of the Indian Ocean and Red Sea (Decelle et al., 2012; Figure 5). This observation suggests that *Phaeocystis* may be more cosmopolitan and diverse than previously thought, and widely dispersed ecotypes of *P. globosa* and *P. cordata* may be present that was previously undiscovered due to under-sampling or limited molecular genetics studies of this species.

The GO-SHIP P15S is a decadal repeat hydrography transect that has previously been conducted several times (Talley et al., 2016). Comparing the chlorophyll *a* profile in this study (Figure 2D) to those from a previous summer occupation of the P15S line (year 1996), peak chlorophyll *a* values were observed in the STZ in both our study and the summer transect, but in the PFZ peak chlorophyll *a* values were only observed in the summer (DiTullio et al., 2003). Using CHEMTAX of algal pigments, DiTullio et al. (2003) detected *Phaeocystis* (as type 4 haptophytes) in the PFZ, STZ and equatorial zone, however, their study did not further differentiate *Phaeocystis* distribution at the species level. In terms of temporal variability in *Phaeocystis* species abundance and distribution, a direct comparison between the earlier P15S summer occupation with this autumn/winter transect would be challenging, since the methods used to classify *Phaeocystis* between the studies were different. The CHEMTAX *Phaeocystis* classification used by DiTullio et al. (2003) was based on the general assumption that *Phaeocystis* expressed more equal ratios of fucoxanthin (fuco), 19′-butanoyloxyfucoxanthin (19but) and 19′-hexanoyloxyfucoxanthin (19hex). This method may not have conclusively determined all *Phaeocystis* since, as mentioned above, *Phaeocystis* are known to express variable pigment composition due to variations in environmental conditions (van Leeuwe and Stefels, 1998; Swan et al., 2016). The method may also have also accounted for other phytoplankton that shared similar diagnostic pigments or missed species of *Phaeocystis* that express different ratios of pigments (Schoemann et al., 2005).

*Phaeocystis* have been previously discovered to form photosymbiotic associations with Acantharia, an abundant open ocean zooplankton, at multiple locations across the global oceans including the Drake Passage sector of the Southern Ocean (Decelle et al., 2012; de Vargas et al., 2015). This has been suggested to be a potentially important ecological adaptation strategy for heterotrophic organisms to maintain their abundance in surface open ocean waters. In our study, Acantharia were only detected at very low relative abundances, ranging between 0.01 and 0.6% of all microbial eukaryotes and were primarily unclassified Acantharia (data not shown). Higher abundances of Acantharia were detected in deeper mesopelagic waters (data not shown). This indicated that Acantharia-*Phaeocystis* photosymbiosis may be a less important mode of adaptation to oligotrophic surface waters in the Southwest Pacific, or that the photosymbiotic association could be seasonally influenced and less prevalent during the austral autumn-winter.

## Conclusion

This high-resolution marker gene survey has shown that *Phaeocystis*, a key summer bloom-forming phytoplankton species within the Southern Ocean is also highly abundant during the autumn-winter months. The high diversity of the *Phaeocystis* genus was strongly partitioned at species level by oceanic fronts acting as ecological boundaries, and the study re-confirmed that the polar and subantarctic fronts are major ecological boundaries in microbial distribution and abundance. *P. globosa* and *P. cordata*, species previously thought to only prevail in the northern hemisphere were detected within this study, indicating that these organisms are more cosmopolitan than previously understood. Further molecular exploration of this genus, particularly in its non-colonial forms should be a key consideration to better understand its seasonal variability, environmental drivers and ecological interactions with other microbes, in efforts to better understand its potential response to climate change and the cascading effects toward the broader marine ecosystem.

## Data Availability Statement

The datasets presented in this study can be found in online repositories. The names of the repository/repositories and accession number(s) can be found in the article/ Supplementary Material.

## Author Contributions

SS collected the samples, planned and executed the experiments, analyzed the data, prepared figures and tables, and wrote the manuscript. LB and TT contributed to the planning of the field program, analyzed and interpreted the data, and reviewed and refined the manuscript.

## Conflict of Interest

The authors declare that the research was conducted in the absence of any commercial or financial relationships that could be construed as a potential conflict of interest.

## Footnotes

1 http://www.go-ship.org

2 http://www.teos-10.org/

3 https://cchdo.ucsd.edu/cruise/096U20160426

4 https://www.ramaciotti.unsw.edu.au/

5 https://www.bioplatforms.com/projects/australian-microbiome/

6 https://data.bioplatforms.com/organization/pages/australian-microbiome/methods

7 https://data.bioplatforms.com/organization/about/australian-microbiome

8 https://www.ncbi.nlm.nih.gov/bioproject/PRJNA385736
