# Supplementary material for: Oceanographic Fronts Shape Phaeocystis Assemblages: A High-Resolution 18S rRNA Gene Survey From the Ice-Edge to the Equator of the South Pacific
Source: Front Microbiol. 2020 Aug 6;11:1847. doi: 10.3389/fmicb.2020.01847 (PMC7424020; doi:10.3389/fmicb.2020.01847)
Supplement: Supplementary file 1 [file Data_Sheet_1.zip › 560928_Sow_AuthorsProof_SuppMat_28072020/Supp_Materials_Phaeocystis_Survey_Frontiers_AllOthers_AuthorProof_28072020.pdf]

Supplementary Material

Oceanographic Fronts Shape *Phaeocystis* Assemblages: A High-Resolution 18S rRNA Gene Survey from the Ice-Edge to the Equator of the South Pacific

Swan L. S. Sow[1,2*], Thomas W. Trull[2] and Levente Bodrossy[2*]

[1] Institute for Marine and Antarctic Studies, University of Tasmania, Hobart, Tasmania, Australia

[2] Oceans and Atmosphere, Commonwealth Scientific and Industrial Research Organisation (CSIRO), Hobart, Tasmania, Australia

*Correspondence: swan.lisan@gmail.com
lev.bodrossy@csiro.au

This file includes:

- Supplementary figures S1-S3
- Supplementary tables S3-S5, S7
- Supplementary methods:
    - DNA extraction protocol
    - List of sequencing primers
    - Protocol for amplification and Illumina sequencing of the 18S rRNA gene
    - Sequence analyses and taxonomic classification methods
- References for supplementary material

Supplementary tables S1, S2 and S6 are in separate files.

1 Supplementary Figures

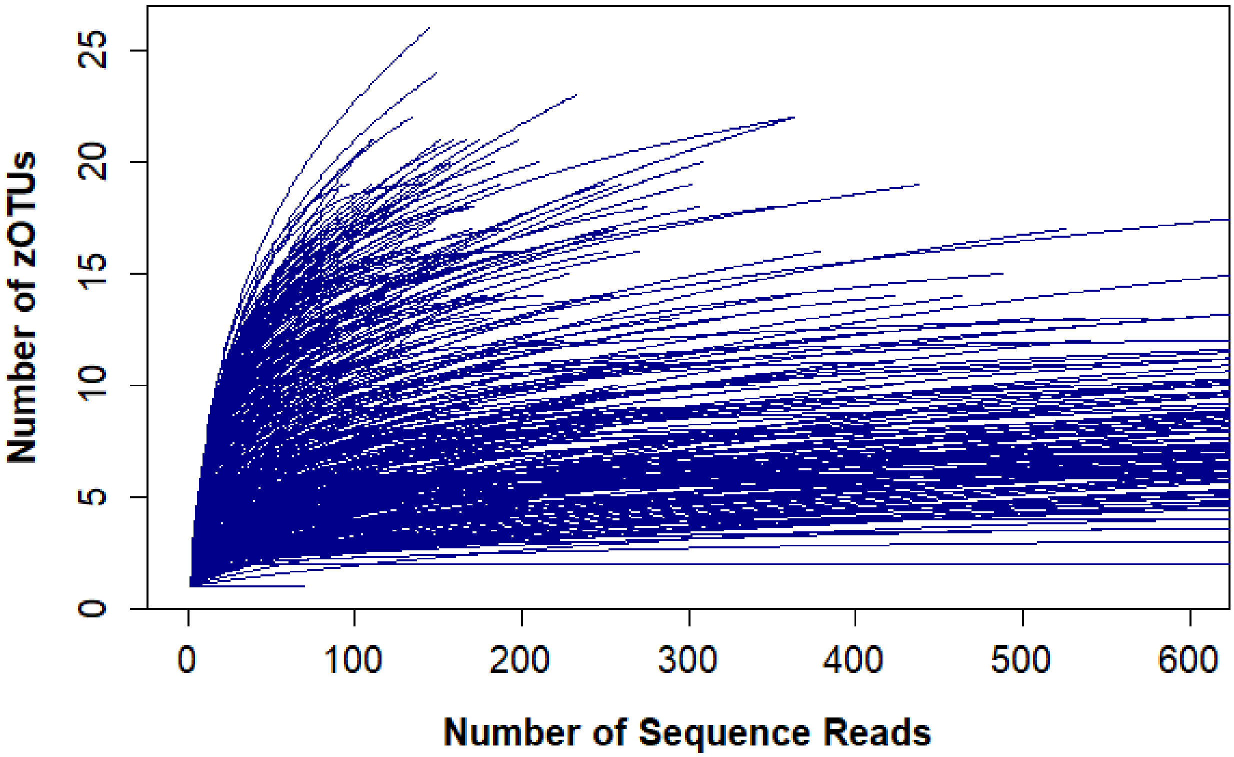

Supplementary Figure S1. Rarefaction curves of the average observed *Phaeocystis* sequences within each sampling station. Samples (including 18S rRNA sequences from all taxa) were subsampled to a depth of 25,000 sequences prior to subsequent analyses.

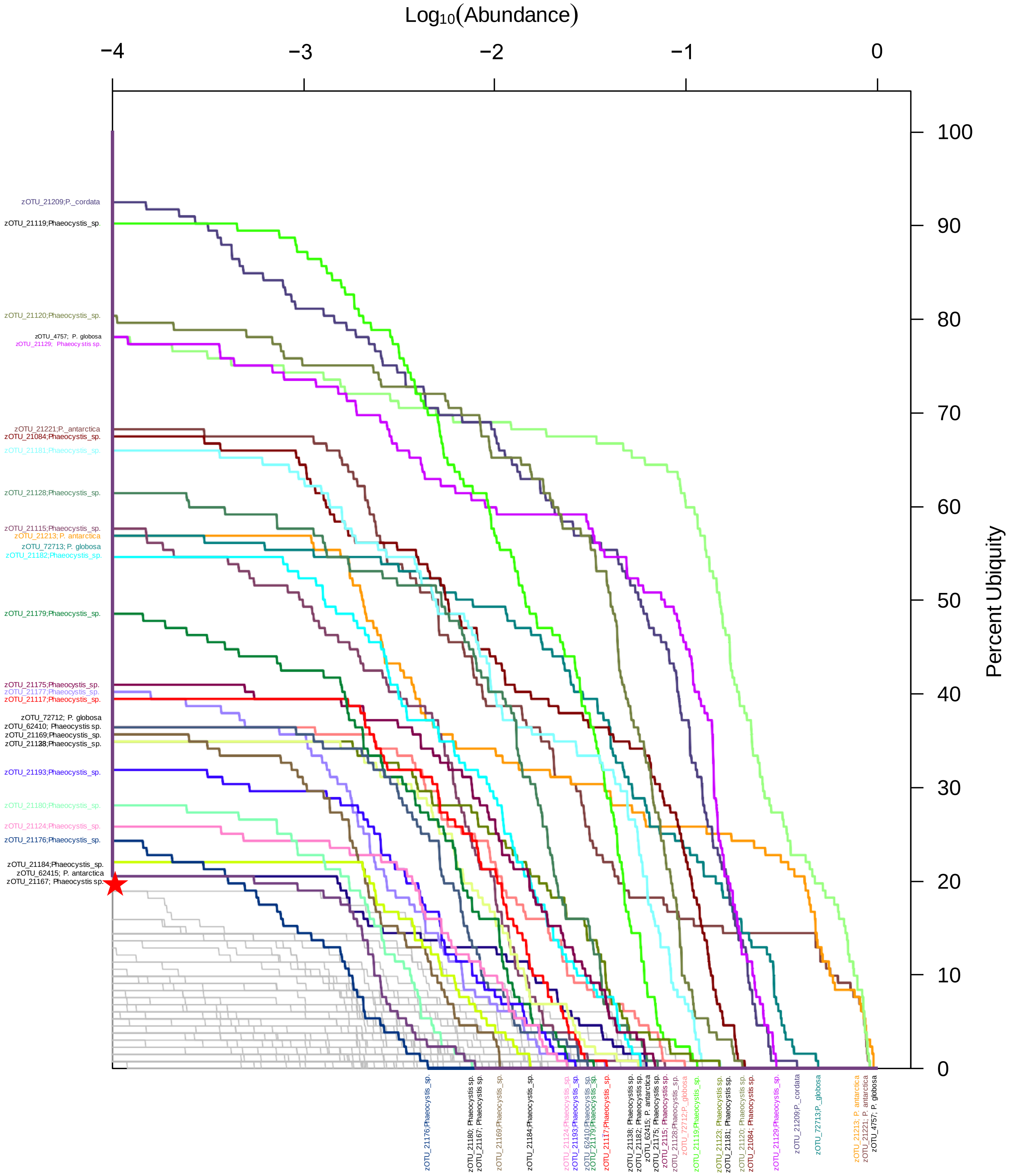

Supplementary Figure S2. Abundance-ubiquity plot for all *Phaeocystis* zOTUs considered within the study. The 0.01%-20 % cumulative relative abundance-ubiquity (Ab-Ub) threshold is indicated by the red star. zOTUs below the Ab-Ub threshold are indicated in gray.

A.

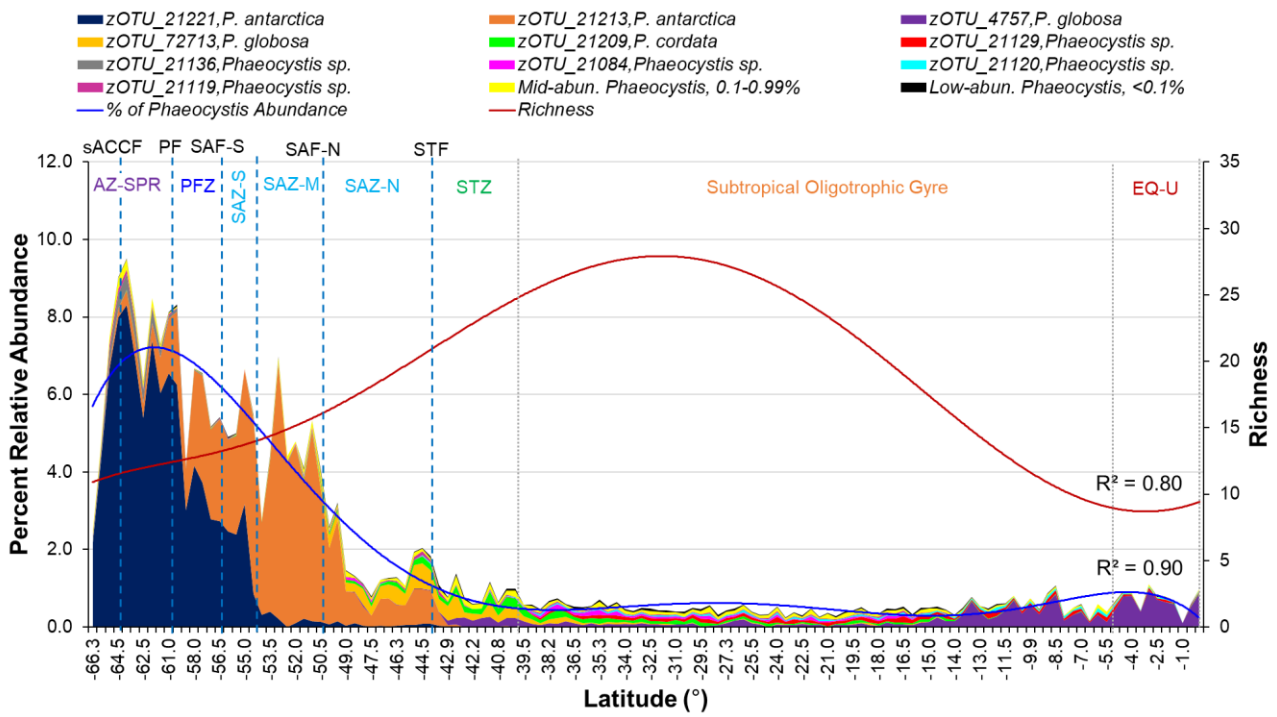

B.

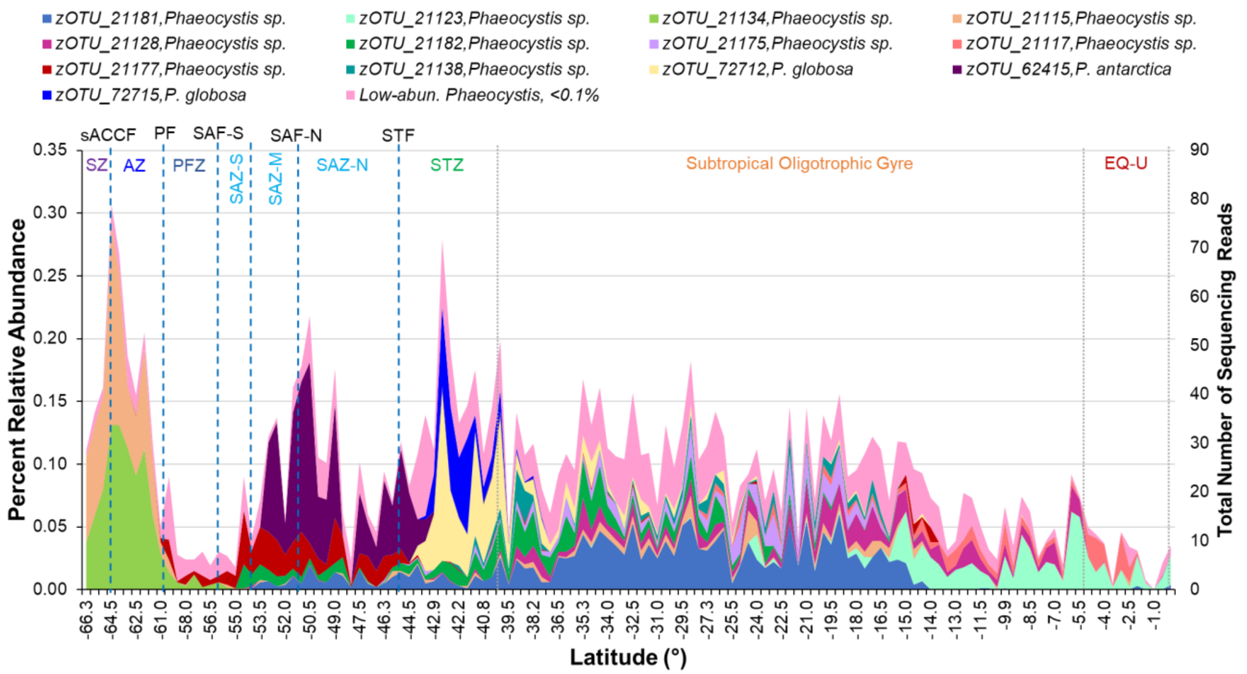

Supplementary Figure S3. **(A)** Percent relative abundance of high-abundance *Phaeocystis* zOTUs (relative abundance > 1% of all *Phaeocystis* 18S rRNA gene sequences) within the P15S transect. All *Phaeocystis* zOTUs above and below the Ab-Ub threshold were considered. Mid- (0.1-0.99% of all *Phaeocystis* 18S rRNA gene sequences) and low- (< 0.1% of all *Phaeocystis* 18S rRNA gene

sequences) abundance zOTUs are indicated as separate, pooled categories. Richness (red line) and percent relative abundance (blue line) profiles of the *Phaeocystis* assemblage fitted with a sixth-order polynomial regression curve are overlaid. (**B)** Average relative abundance of mid-abundance *Phaeocystis* zOTUs. All *Phaeocystis* zOTUs above and below the Ab-Ub threshold were considered. Low abundance zOTUs are indicated as a pooled category. Approximate locations of fronts are indicated by blue dotted lines, while approximate boundaries for subtropical gyre and equatorial upwelling zones are indicated by grey dotted lines. Acronyms for fronts and zones are as listed in Figure 1.

2 Supplementary Tables

Supplementary Table S3. Percentage relative abundances and ubiquity of the 29 *Phaeocystis* zOTUs above the 0.01%-20% Ab-Ub threshold identified within the P15S transect. The percentages indicated were calculated as total cumulative percentages for each zOTU of the total *Phaeocystis* sequences, as well as percentages for each zOTU of the total 18S rRNA gene sequences of all samples considered within this section of the study. 'high', 'mid' and 'low' abundance categories were determined based on the percentage of each zOTU out of total *Phaeocystis* sequences.

	**zOTU Id**	**Taxonomy**	**Proportion out of Total *Phaeocystis* (%)**	**Proportion out of Total 18S rRNA gene sequences (%)**	**Ubiquity (%)**
**High Abundance, > 1%**	zOTU_21221	*Phaeocystis antarctica*	40.15	0.726	68.2
	zOTU_21213	*Phaeocystis antarctica*	32.05	0.580	56.8
	zOTU_4757	*Phaeocystis globosa*	8.22	0.149	78.0
	zOTU_72713	*Phaeocystis globosa*	3.95	0.071	56.8
	zOTU_21209	*Phaeocystis cordata*	2.94	0.053	92.4
	zOTU_21129	*Phaeocystis* sp.	2.37	0.043	78.0
	zOTU_21084	*Phaeocystis* sp.	1.28	0.023	67.4
	zOTU_21120	*Phaeocystis* sp.	1.24	0.022	80.3
	zOTU_21119	*Phaeocystis* sp.	1.23	0.022	90.2
**Mid Abundance, 0.1-0.99%**	zOTU_21181	*Phaeocystis* sp.	0.73	0.013	65.9
	zOTU_62415	*Phaeocystis_antarctica*	0.47	0.009	20.5
	zOTU_21115	*Phaeocystis* sp.	0.42	0.008	57.6
	zOTU_72712	*Phaeocystis_globosa*	0.35	0.006	36.4
	zOTU_21123	*Phaeocystis* sp.	0.31	0.006	34.8
	zOTU_21128	*Phaeocystis* sp.	0.31	0.006	61.4
	zOTU_21182	*Phaeocystis* sp.	0.29	0.005	54.5
	zOTU_21177	*Phaeocystis* sp.	0.22	0.004	40.2
	zOTU_21175	*Phaeocystis* sp.	0.20	0.004	40.9
	zOTU_21117	*Phaeocystis* sp.	0.14	0.003	39.4
	zOTU_21138	*Phaeocystis* sp.	0.11	0.002	34.8
**Low Abund., < 0.1%**	zOTU_21179	*Phaeocystis* sp.	0.09	0.0016	48.5
	zOTU_62410	*Phaeocystis* sp.	0.09	0.0016	36.4
	zOTU_21193	*Phaeocystis* sp.	0.06	0.0011	31.8
	zOTU_21124	*Phaeocystis* sp.	0.06	0.0011	25.8
	zOTU_21169	*Phaeocystis* sp.	0.04	0.0006	35.6
	zOTU_21184	*Phaeocystis* sp.	0.03	0.0006	22.0
	zOTU_21180	*Phaeocystis* sp.	0.03	0.0006	28.0
	zOTU_21176	*Phaeocystis* sp.	0.03	0.0005	24.2
	zOTU_21167	*Phaeocystis* sp.	0.02	0.0003	20.5

Supplementary Table S4. Pairwise Analysis of Similarities (ANOSIM) test for unordered *Phaeocystis* assemblage differences between oceanographic zones in this study. ANOSIM included only the 29 zOTUs above the 0.01%-20% Ab-Ub threshold listed in Supplementary Table S3 and Supplementary Figure S2. Acronyms for oceanographic zones are as detailed in Figure 1. Number of permutations: 9999.

**Oceanographic Zone Pair**	**R Statistic**	**Significance Level (%)**
AZ-SPR, PFZ	0.937	0.01
AZ-SPR, SAZ-S	0.994	0.3
AZ-SPR, SAZ-M	1	0.01
AZ-SPR, SAZ-N	1	0.01
AZ-SPR, STZ	1	0.02
AZ-SPR, STOG	1	0.01
AZ-SPR, EQ-U	1	0.01
PFZ, SAZ-S	0.608	2.4
PFZ, SAZ-M	1	0.02
PFZ, SAZ-N	1	0.01
PFZ, STZ	1	0.02
PFZ, STOG	1	0.01
PFZ, EQ-U	1	0.01
SAZ-S, SAZ-M	0.655	0.8
SAZ-S, SAZ-N	0.989	0.2
SAZ-S, STZ	1	0.6
SAZ-S, STOG	1	0.01
SAZ-S, EQ-U	1	0.3
SAZ-M, SAZ-N	0.843	0.01
SAZ-M, STZ	0.999	0.02
SAZ-M, STOG	0.999	0.01
SAZ-M, EQ-U	1	0.01
SAZ-N, STZ	0.9	0.01
SAZ-N, STOG	0.892	0.01
SAZ-N, EQ-U	1	0.01
STZ, STOG	0.505	0.01
STZ, EQ-U	1	0.01
STOG, EQ-U	0.514	0.01

Supplementary Table S5. Pairwise Analysis of Similarities (ANOSIM) test for unordered *Phaeocystis* assemblage differences between oceanographic zones in this study. ANOSIM include all zOTUs classified as the genus *Phaeocystis*. Acronyms for oceanographic zones are as detailed in Figure 1. Number of permutations: 9999.

**Oceanographic Zone Pair**	**R Statistic**	**Significance Level (%)**
AZ-SPR, PFZ	0.951	0.01
AZ-SPR, SAZ-S	0.997	0.3
AZ-SPR, SAZ-M	1	0.01
AZ-SPR, SAZ-N	1	0.01
AZ-SPR, STZ	1	0.01
AZ-SPR, STOG	1	0.01
AZ-SPR, EQ-U	1	0.01
PFZ, SAZ-S	0.656	1.8
PFZ, SAZ-M	1	0.02
PFZ, SAZ-N	1	0.01
PFZ, STZ	1	0.02
PFZ, STOG	1	0.01
PFZ, EQ-U	1	0.01
SAZ-S, SAZ-M	0.647	0.8
SAZ-S, SAZ-N	0.988	0.2
SAZ-S, STZ	1	0.6
SAZ-S, STOG	1	0.01
SAZ-S, EQ-U	1	0.3
SAZ-M, SAZ-N	0.826	0.01
SAZ-M, STZ	0.998	0.02
SAZ-M, STOG	0.999	0.01
SAZ-M, EQ-U	1	0.01
SAZ-N, STZ	0.934	0.01
SAZ-N, STOG	0.888	0.01
SAZ-N, EQ-U	1	0.01
STZ, STOG	0.562	0.01
STZ, EQ-U	1	0.01
STOG, EQ-U	0.465	0.01

3 Supplementary Methods

3.1 DNA Extraction

DNeasy® PowerWater® Sterivex™ extraction kit protocol as modified by Appleyard et al. (2013)

Materials:

Lysis buffer stock solutions

200 mM $NaH_2PO_4 2H_2O$ (monobasic) (MW=156, 156/1 L = 1M, 6.24/200 mL = 200 mM in 200 mL solution)

200 mM Na_2HPO_4 (dibasic) (MW=142, 142 g/1 L =1M, 5.68 g/200 mL = 200 mM in 200 mL solution)

To make up 200 mL lysis buffer:

39 mL 200 mM NaH_2PO_4 (monobasic)
61 mL 200 mM Na_2HPO_4 (dibasic)
17.54 g NaCl
2 g CTAB
4 g PVP K30

Add distilled, deionized water to make up to 200 mL
Adjust to pH 7.0 (using NaOH – try couple of mL of 10 M NaOH)

From FastDNA™ Spin Kit for Soil (MP Biomedicals):
MT buffer

From DNeasy® PowerWater® Sterivex™ Kit (Qiagen):
Columns and sample recovery tubes, 3 mL and 20 mL syringes, 5 mL tubes
Solution MR (contains 3 M guanidinium thiocyanate high concentration salt solution) (warmed to 65°C before use)
Ethanol
Solution PW (contains ethanol)
Inlet and outlet caps for Sterivex™ filters

Lysozyme
Proteinase K – 20 mg/mL
Phenol:Chloroform:Isoamyl (25:24:1) (PCI)
Chloroform:Isoamyl (24:1) (CI)
TE buffer

Protocol:

1. Weigh 125 mg lysozyme into 50 mL falcon tube and add 25 mL Lysis Buffer to dissolve (lysozyme final concentration 5 mg/mL).
2. Remove filters from -80°C, remove inlet cap and using a pipette add 1.875 mL Lysis buffer (containing 5mg/mL final concentration of lysozyme) and 0.125 mL MT buffer.
3. Recap the Sterivex™ filter and attach filter (with inlet end facing out) to the horizontal vortexer, speed 5-7 for 60 min (turning the filter a couple of times during the hour).

4. Using 3 mL syringe, draw back plunger and attach to inlet end of filter until pressure builds up – release plunger and buffer in filter should flow into syringe. Divide approximately 2 mL of buffer evenly into 2 × 2.0 mL tubes (do not use the 2 mL collection tubes that come in the PowerWater® kit – they don't tolerate PCI) (may need to use syringe several times to get all buffer out of filter, should be about 0.800-1.00 mL per tube).
5. In fume hood, add 900 µL PCI to each tube, invert several times, spin down 13000 rpm for 10 mins at room temperature.
6. Combine the aqueous phases from both tubes into one 2.0 mL tube (which will be between 1.2 – 1.5 mL), add 20 µL Proteinase K, onto heat block for 2 hours at 60°C.
7. In fume hood, add 500 µL CI, spin down 13000 rpm for 10 mins at room temperature – put aqueous phase into new tube.
8. In fume hood, add a further 500 µL CI, spin down 13000 rpm for 5 mins at room temperature – put aqueous phase into new tube.
9. After the second spin, take out 1 mL of aqueous phase, add to 5 ml tube.
10. Add 3 mL of warmed Solution MR (65°C), mix by inversion.
11. Attach column to barrel of 20 mL syringe and attach to vacuum manifold.
12. Pour contents of 5 mL tube into barrel while still warm.
13. Using vacuum, pull contents through the column.
14. While keeping column attached to the manifold, remove barrel and add 800 µL ethanol to column. Using vacuum, pull contents through the column.
15. Add 800 µL Solution PW to column. Using vacuum, pull contents through the column, then keep on vacuum for 2 mins.
16. Turn vacuum off, put column into new 2.0 mL tube and let air dry on bench for 10 mins
17. Add 80 µL 0.1× TE buffer to column, incubate at 37°C for 45 min.
18. Spin down column and tube at 13000 rpm for 2 mins at room temperature to elute DNA.

3.2 Sequencing Primers

Supplementary Table S7: Primer pair sequences used for the tag-sequencing assays targeting the 18S rRNA gene of *Phaeocystis* within this study.

Primer Name	Primer Sequence
18SV4F (TAReuk454FWD1)	CCAGCASCYGCGGTAATTCC
18SV4R (TAReuk- Rev3)	ACTTTCGTTCTTGATYRATGATCTRYATC

3.3 Amplification and Illumina Sequencing of the V4 region of the 18S rRNA gene

Please see the next page. This protocol was directly extracted from: https://data.bioplatforms.com/organization/pages/bpa-marine-microbes/methods

Ramaciotti Centre for Genomics

Amplification and Illumina Sequencing of the V4 region of the 18S rRNA gene

1.0 Introduction

The protocol detailed here is designed to amplify the V4 region of the 18S rRNA gene for paired-end 18S community sequencing on the Illumina MiSeq platform. This protocol is based on Illumina's 16S Metagenomic Sequencing Library Preparation guide and the protocol used by Ocean Sampling Day, modified to amplify the target and add indexed adapter sequences in a single PCR step.

2.0 Amplification of the V4 region of the 18S rRNA gene

2.1 Primers for amplification of V4 region of the 18S rRNA gene

Amplification primers
See Appendix 1 for full list of primer sequences.

Forward primer
Field number (space-delimited), description:
5' Illumina adapter
Nextera XT i5 index sequence
Illumina forward overhang sequence
18S V4 forward

AATGATACGGCGACCACCGAGATCTACAC XXXXXXXX TCGTCGGCAGCGTCAGATGTGTATAAGAGACAG CCAGCASCYGCGGTAATTCC

Reverse primer
Field number (space-delimited), description:
Reverse complement of 3' Illumina adapter
Nextera XT i7 index sequence
Illumina reverse overhang sequence
18S V4 reverse

CAAGCAGAAGACGGCATACGAGAT XXXXXXXX GTCTCGTGGGCTCGGAGATGTGTATAAGAGACAG ACTTTCGTTCTTGATYRATGA

2.2 Master mix for amplification of V4 region of the 18S rRNA gene

Component	Volume 1 rxn
KAPA HiFi Hot Start Readymix (2x) [a]	12.5
H_2O	9
Forward primer (10 µm)	1.25
Reverse primer (10 µm)	1.25
Template	1
**Total Volume**	**25**

Kit code KK2601 or KK2602

2.3 Thermocycler Conditions for amplification of V4 region of the 18S rRNA gene (96 well thermocyclers)

	Temperature	Time (mm:ss)
Activation	98°C	0:30
Amplification (10 cycles)	98°C	0:10
	44°C	0:30
	72°C	0:15
Amplification (20 cycles)	98°C	0:10
	62°C	0:30
	72°C	0:15
Final Extension	72°C	7:00
HOLD	4°C	∞

2.4 Process

2.4.1 Use undiluted DNA as a first attempt, and 1:10 diluted for repeats/failed reactions
2.4.2 Amplify samples with conditions outlined above.
2.4.3 Run amplicons on an agarose gel. Expected band size for 18S-V4 is approximately 536bp.
2.4.4 Clean and normalize the PCR products using SequalPrep Normalization plates according to manufacturer's instructions (Invitrogen cat no. A10510-01)
2.4.5 Pool equal volumes of each normalized amplicon.
2.4.6 Perform QC on pool using Qubit (concentration) and Tapestation (size) and calculate molarity of pool.

3.0 Sequencing of V4 region of the 18S rRNA gene

3.1 Sequencing Setup

3.2 Dilute pool prepared in **step 2.4.6** to **4nM**.
3.3 Denature according to Ilumina protocol. See *Preparing Libraries for Sequencing on the MiSeq (part #15039740).*
3.4 Prepare MiSeq Reagent Cartridge (v2 500-cycles). See *MiSeq Reagent Kit v2 - Reagent Preparation Guide* (part # 15034097).
3.5 Load 600 µl of library pool into the MiSeq reagent cartridge in designated reservoir
3.6 Prepare sample sheet to include the appropriate index sequences.
3.7 Start sequencing run following *MiSeq System User Guide (part # 15027617).*

References

16S Metagenomic Sequencing Library Preparation (Illumina Part # 15044223 Rev. B) available here: http://www.illumina.com/content/dam/illumina-support/documents/documentation/chemistry_documentation/16s/16s-metagenomic-library-prep-guide-15044223-b.pdf
LifeWatch Italy Ocean Sampling Day 2014 Protocol – available here: http://mb3is.megx.net/osd-files/download?path=/2014/protocols&files=OSD2014_protocol_B_18S_V4andV9_Sequencing_LifeWath_MoBiLab_BARI.pdf

Appendix 1. Primer Sequences

Primer Name	Primer Sequence
18S-V4f_S502	AATGATACGGCGACCACCGAGATCTACACCTCTCTATTCGTCGGCAGCGTCAGATGTGTATAAGAGACAGCCAGCASCYGCGGTAATTCC
18S-V4f_S503	AATGATACGGCGACCACCGAGATCTACACTATCCTCTTCGTCGGCAGCGTCAGATGTGTATAAGAGACAGCCAGCASCYGCGGTAATTCC
18S-V4f_S505	AATGATACGGCGACCACCGAGATCTACACGTAAGGAGTCGTCGGCAGCGTCAGATGTGTATAAGAGACAGCCAGCASCYGCGGTAATTCC
18S-V4f_S506	AATGATACGGCGACCACCGAGATCTACACACTGCATATCGTCGGCAGCGTCAGATGTGTATAAGAGACAGCCAGCASCYGCGGTAATTCC
18S-V4f_S507	AATGATACGGCGACCACCGAGATCTACACAAGGAGTATCGTCGGCAGCGTCAGATGTGTATAAGAGACAGCCAGCASCYGCGGTAATTCC
18S-V4f_S508	AATGATACGGCGACCACCGAGATCTACACCTAAGCCTTCGTCGGCAGCGTCAGATGTGTATAAGAGACAGCCAGCASCYGCGGTAATTCC
18S-V4f_S510	AATGATACGGCGACCACCGAGATCTACACCGTCTAATTCGTCGGCAGCGTCAGATGTGTATAAGAGACAGCCAGCASCYGCGGTAATTCC
18S-V4f_S511	AATGATACGGCGACCACCGAGATCTACACTCTCTCCGTCGTCGGCAGCGTCAGATGTGTATAAGAGACAGCCAGCASCYGCGGTAATTCC
18S-V4f_S513	AATGATACGGCGACCACCGAGATCTACACTCGACTAGTCGTCGGCAGCGTCAGATGTGTATAAGAGACAGCCAGCASCYGCGGTAATTCC
18S-V4f_S515	AATGATACGGCGACCACCGAGATCTACACTTCTAGCTTCGTCGGCAGCGTCAGATGTGTATAAGAGACAGCCAGCASCYGCGGTAATTCC
18S-V4f_S516	AATGATACGGCGACCACCGAGATCTACACCCTAGAGTTCGTCGGCAGCGTCAGATGTGTATAAGAGACAGCCAGCASCYGCGGTAATTCC
18S-V4f_S517	AATGATACGGCGACCACCGAGATCTACACGCGTAAGATCGTCGGCAGCGTCAGATGTGTATAAGAGACAGCCAGCASCYGCGGTAATTCC
18S-V4f_S518	AATGATACGGCGACCACCGAGATCTACACCTATTAAGTCGTCGGCAGCGTCAGATGTGTATAAGAGACAGCCAGCASCYGCGGTAATTCC
18S-V4f_S520	AATGATACGGCGACCACCGAGATCTACACAAGGCTATTCGTCGGCAGCGTCAGATGTGTATAAGAGACAGCCAGCASCYGCGGTAATTCC
18S-V4f_S521	AATGATACGGCGACCACCGAGATCTACACGAGCCTTATCGTCGGCAGCGTCAGATGTGTATAAGAGACAGCCAGCASCYGCGGTAATTCC
18S-V4f_S522	AATGATACGGCGACCACCGAGATCTACACTTATGCGATCGTCGGCAGCGTCAGATGTGTATAAGAGACAGCCAGCASCYGCGGTAATTCC
18S-V4Lr_N701	CAAGCAGAAGACGGCATACGAGATTCGCCTTAGTCTCGTGGGCTCGGAGATGTGTATAAGAGACAGACTTTCGTTCTTGATYRATGA
18S-V4Lr_N702	CAAGCAGAAGACGGCATACGAGATCTAGTACGGTCTCGTGGGCTCGGAGATGTGTATAAGAGACAGACTTTCGTTCTTGATYRATGA
18S-V4Lr_N703	CAAGCAGAAGACGGCATACGAGATTTCTGCCTGTCTCGTGGGCTCGGAGATGTGTATAAGAGACAGACTTTCGTTCTTGATYRATGA
18S-V4Lr_N704	CAAGCAGAAGACGGCATACGAGATGCTCAGGAGTCTCGTGGGCTCGGAGATGTGTATAAGAGACAGACTTTCGTTCTTGATYRATGA
18S-V4Lr_N705	CAAGCAGAAGACGGCATACGAGATAGGAGTCCGTCTCGTGGGCTCGGAGATGTGTATAAGAGACAGACTTTCGTTCTTGATYRATGA
18S-V4Lr_N706	CAAGCAGAAGACGGCATACGAGATCATGCCTAGTCTCGTGGGCTCGGAGATGTGTATAAGAGACAGACTTTCGTTCTTGATYRATGA
18S-V4Lr_N707	CAAGCAGAAGACGGCATACGAGATGTAGAGAGGTCTCGTGGGCTCGGAGATGTGTATAAGAGACAGACTTTCGTTCTTGATYRATGA
18S-V4Lr_N710	CAAGCAGAAGACGGCATACGAGATCAGCCTCGGTCTCGTGGGCTCGGAGATGTGTATAAGAGACAGACTTTCGTTCTTGATYRATGA
18S-V4Lr_N711	CAAGCAGAAGACGGCATACGAGATTGCCTCTTGTCTCGTGGGCTCGGAGATGTGTATAAGAGACAGACTTTCGTTCTTGATYRATGA
18S-V4Lr_N712	CAAGCAGAAGACGGCATACGAGATTCCTCTACGTCTCGTGGGCTCGGAGATGTGTATAAGAGACAGACTTTCGTTCTTGATYRATGA
18S-V4Lr_N714	CAAGCAGAAGACGGCATACGAGATTCATGAGCGTCTCGTGGGCTCGGAGATGTGTATAAGAGACAGACTTTCGTTCTTGATYRATGA
18S-V4Lr_N715	CAAGCAGAAGACGGCATACGAGATCCTGAGATGTCTCGTGGGCTCGGAGATGTGTATAAGAGACAGACTTTCGTTCTTGATYRATGA
18S-V4Lr_N716	CAAGCAGAAGACGGCATACGAGATTAGCGAGTGTCTCGTGGGCTCGGAGATGTGTATAAGAGACAGACTTTCGTTCTTGATYRATGA
18S-V4Lr_N718	CAAGCAGAAGACGGCATACGAGATGTAGCTCCGTCTCGTGGGCTCGGAGATGTGTATAAGAGACAGACTTTCGTTCTTGATYRATGA
18S-V4Lr_N719	CAAGCAGAAGACGGCATACGAGATTACTACGCGTCTCGTGGGCTCGGAGATGTGTATAAGAGACAGACTTTCGTTCTTGATYRATGA
18S-V4Lr_N720	CAAGCAGAAGACGGCATACGAGATAGGCTCCGGTCTCGTGGGCTCGGAGATGTGTATAAGAGACAGACTTTCGTTCTTGATYRATGA
18S-V4Lr_N721	CAAGCAGAAGACGGCATACGAGATGCAGCGTAGTCTCGTGGGCTCGGAGATGTGTATAAGAGACAGACTTTCGTTCTTGATYRATGA
18S-V4Lr_N722	CAAGCAGAAGACGGCATACGAGATCTGCGCATGTCTCGTGGGCTCGGAGATGTGTATAAGAGACAGACTTTCGTTCTTGATYRATGA
18S-V4Lr_N723	CAAGCAGAAGACGGCATACGAGATGAGCGCTAGTCTCGTGGGCTCGGAGATGTGTATAAGAGACAGACTTTCGTTCTTGATYRATGA
18S-V4Lr_N724	CAAGCAGAAGACGGCATACGAGATCGCTCAGTGTCTCGTGGGCTCGGAGATGTGTATAAGAGACAGACTTTCGTTCTTGATYRATGA
18S-V4Lr_N726	CAAGCAGAAGACGGCATACGAGATGTCTTAGGGTCTCGTGGGCTCGGAGATGTGTATAAGAGACAGACTTTCGTTCTTGATYRATGA
18S-V4Lr_N727	CAAGCAGAAGACGGCATACGAGATACTGATCGGTCTCGTGGGCTCGGAGATGTGTATAAGAGACAGACTTTCGTTCTTGATYRATGA
18S-V4Lr_N728	CAAGCAGAAGACGGCATACGAGATTAGCTGCAGTCTCGTGGGCTCGGAGATGTGTATAAGAGACAGACTTTCGTTCTTGATYRATGA
18S-V4Lr_N729	CAAGCAGAAGACGGCATACGAGATGACGTCGAGTCTCGTGGGCTCGGAGATGTGTATAAGAGACAGACTTTCGTTCTTGATYRATGA

3.4 Sequence Analyses and Taxonomic Classification

The quality of the paired reads were evaluated using FastQC v0.11.5 (Andrews, 2014), then trimmed and merged using FLASH v1.2.11 (Magoc and Salzberg, 2011). Quality filtered sequences are mapped to biologically correct, chimera-free zOTUs using USEARCH 64-bit v10.0.240 (Edgar, 2010) and a sample by read abundance matrix is generated. For detailed, step-by-step protocol of DNA sequence processing and analyses, we refer the reader to Brown et al. (2018).

Eukaryote zOTUs were taxonomically identified with the Protist Ribosomal Reference Database (PR^2, v4.11.1) (Guillou et al., 2013) using MOTHUR's implementation of the Wang classifier with a 60% Bayesian probability cut-off (Wang et al., 2007;Schloss et al., 2009). Sequences that were unidentified or incorrectly identified at the kingdom or phylum level were removed from the abundance matrix prior to further analysis.

4 References:

Andrews, S. (2014). *FastQC: a quality control tool for high throughput sequence data*. http://www.bioinformatics.babraham.ac.uk/projects/fastqc/

Appleyard, S., Abell, G., and Watson, R. (2013). "Tackling microbial related issues in cultured shellfish via integrated molecular and water chemistry approaches.", in: *Seafood CRC Final Report (2011/729).*).

Brown, M.V., Van De Kamp, J., Ostrowski, M., Seymour, J.R., Ingleton, T., Messer, L.F., et al. (2018). Systematic, continental scale temporal monitoring of marine pelagic microbiota by the Australian Marine Microbial Biodiversity Initiative. *Scientific Data* 5**,** 180130.

Edgar, R.C. (2010). Search and clustering orders of magnitude faster than BLAST. *Bioinformatics* 26**,** 2460-2461.

Guillou, L., Bachar, D., Audic, S., Bass, D., Berney, C., Bittner, L., et al. (2013). The Protist Ribosomal Reference database (PR(2)): a catalog of unicellular eukaryote Small Sub-Unit rRNA sequences with curated taxonomy. *Nucleic Acids Research* 41**,** D597-D604.

Magoc, T., and Salzberg, S.L. (2011). FLASH: fast length adjustment of short reads to improve genome assemblies. *Bioinformatics* 27**,** 2957-2963.

Schloss, P.D., Westcott, S.L., Ryabin, T., Hall, J.R., Hartmann, M., Hollister, E.B., et al. (2009). Introducing mothur: Open-Source, Platform-Independent, Community-Supported Software for Describing and Comparing Microbial Communities. *Applied and Environmental Microbiology* 75**,** 7537-7541.

Wang, Q., Garrity, G.M., Tiedje, J.M., and Cole, J.R. (2007). Naïve Bayesian Classifier for Rapid Assignment of rRNA Sequences into the New Bacterial Taxonomy. *Applied and Environmental Microbiology* 73**,** 5261-5267.
